# Assessing strategies to mitigate phosphorus leaching from drained clay soils

**DOI:** 10.1007/s13280-017-0991-x

**Published:** 2017-11-20

**Authors:** Barbro Ulén, Mats Larsbo, Johannes Koestel, Qarin Hellner, Maria Blomberg, Pia Geranmayeh

**Affiliations:** Department of Soil and Environment, Swedish University of Agricultural Sciences, P.O. Box 7014, 75007 Uppsala, Sweden

**Keywords:** Drain renovation, Hydrological index, Lime-filter ditch, Soil tillage

## Abstract

Assessing mitigation of phosphorus (P) leaching from subsurface drainage systems is challenging due to high spatial and temporal variation in leaching. Mean measured total P leaching from a clayey soil in an eight-year study period (four replicates per treatment) was (kg ha^−1^ year^−1^): 1.21 from shallow autumn tillage (ShT), 0.84 from unfertilised fallow (UF), 0.81 from conventional autumn ploughing (CT) and 0.57 from structure liming (SL–CT). Treatment was not significant using Richards–Baker flow index or a distance factor as covariate (*p* = 0.084 and 0.057). A tendency for lower leaching was obtained comparing SL-CT with ShT (*p*_adjusted_ = 0.060 and 0.009 respectively). A combination of measures adapted to drainage conditions and clay content in different parts of the field is proposed since P leaching was approximately halved from an adjacent field (4.3 ha) in a three-year post-period compared with a three-year pre-period for structure liming the entire field and drainage system renovation plus structure lime drain backfilling.

## Introduction

Mitigating phosphorus (P) leaching from arable land is critical for improving water quality and reducing undesirable eutrophication of lakes and seas. However, P leaching is known to vary widely in time and space (e.g. Haygarth et al. 2005), hampering assessment of efforts to reduce leaching. In general, agricultural practices affect P leaching less than meteorological conditions, but P transport through the soil is also strongly dependent on soil structure (Jarvis 2007). Since soil structure may vary considerably within fields, high in-field variation in P leaching has been reported (e.g. Norgaard et al. 2013).

In Sweden, 70% of arable land is artificially drained (Elmquist 2014). An efficient drainage system can alter water quality through changes in hydrology and stabilisation of groundwater level (Skaggs et al. 1994). Dilution of solutes from the topsoil can occur when shallow groundwater is mixed in drains with infiltrating water from the soil surface. This was demonstrated in a previous 9-years’ study on 16 fields within a 1-km radius that were all tile-drained to improve infiltration of precipitation (Prec) water into the soil (Ulén et al. 2016). All fields were assumed to receive the same yearly precipitation, but measured discharge (*Q*, mm year^−1^) at the drain outlets varied widely between fields, probably owing to different contributions of shallow groundwater. This dilution resulted in a significant negative correlation between concentrations of dissolved reactive P (DRP) and *Q*/Prec ratio. Since yearly (flow-proportionally) total P (TP) concentration was not significantly correlated to discharge, *Q* could not be used as a predictor for TP leaching from the drainage system (Ulén et al. 2016).

To improve infiltration and reduce P losses from arable land, installation/renovation of drainage systems is very important (Taylor et al. 2016). To further improve infiltration, quicklime (CaO), also known as burnt lime, can be added to drain backfill (Lindström and Ulén 2003). This measure can reduce surface runoff, with an accompanying reduction in eroded soil and attached P. In studies in Lithuania, addition of burnt shell-ash (CaO, at 0.6% of soil mass) to drain backfill has been tested for reducing P losses (Šaulys and Bastienė 2007). Apart from such lime-filter ditches, structure liming (applying quicklime or slaked (hydrated) lime to the entire topsoil) can improve water infiltration over the whole field area, thereby reducing the risk of surface water ponding. Surface ponding (e.g. in micro-depressions) can increase non-equilibrium water flow and solute transport in macropores (Jarvis 2007). Macropore flow which generally leads to a fast response in tile drains with increased water flow peak flows has been suggested to be related to a high flashiness (Deelstra and Iital 2008). In contrast, contributions by shallow groundwater can damp such peaks and modify water flow fluctuations since the shallow groundwater level fluctuates much slower (e.g. Beven and Gerdman 2013).

A range of hydrological indices for flow alteration are available, considering, e.g. variation in flows (flashiness), frequency and duration of flow peaks and flow skewness. The Richards–Baker flashiness index (Baker et al. 2004) is commonly based on daily time steps (FI_day_). This relative simple index has been used as an explanatory factor for nutrient leaching in a range of agricultural catchments (Deelstra et al. 2014). At farm scale, a significant relationship between FI_day_ and leaching of total P (TP) has been reported (Ulén et al. 2016). Concentration of topsoil P, extracted in acid ammoniumlactate (P-AL) according to Egnér et al. (1960), was found to be another important factor in that nine-year study, whereas annual agricultural management, e.g. tillage, crop and fertilisation, did not affect TP leaching significantly for different fields (Ulén et al. 2016).

High spatial variation in both P and pesticide leaching was observed in a field plot experiment with two rows of 14 drained plots running towards a ditch at the centre of a flat valley (Ulén et al. 2013). Phosphorus and pesticide concentrations in drain water decreased with increasing distance from the ditch (Ulén et al. 2013). Accordingly, this distance was used as a predictor (covariate) in a first six-year assessment of P leaching mitigation strategies at the site (Svanbäck et al. 2014). However, differences in drain flow dynamics between plots were not considered. In the present study, we therefore tested the Richards–Baker index as an alternative and potentially more generally applicable indicator of P leaching, using results from an extended period (eight years) at the same site. A time resolution of one hour (FI_hour_) was chosen to match the small area of the experimental plots, for which peak duration are commonly less than one day. Moreover, leaching of TP and particles together with FI_hour_ was estimated for a nearby field with similar soil type, where improved drainage system in the most crucial parts of the field possibly affected water infiltration and water flow. Previous monitoring of P leaching from the field showed that yearly losses were unaffected by crop, fertilisation and soil tillage (Ulén and Persson 1999).

The overall objective of this study was to assess yearly P leaching via drain systems from experimental plots for a prolonged period with four different management systems considering variation in flows. A second objective was to evaluate the mitigation of P leaching in a nearby field where structure lime (in a common commercial form with slaked lime mixed with milled limestone) had been applied to the topsoil of the entire field area. Additionally, the tile drainage system had been renovated and structure lime used as a backfill above drains in the middle and lower part of the field. A specific objective was to assess if FI_hour_ could be used as an alternative predictor for P concentrations in drainage water to a previous used local factor—the distance to the receiving ditch in the valley.

## Materials and methods

### Site description

#### The Oxelby experimental site

**Fig. 1:**
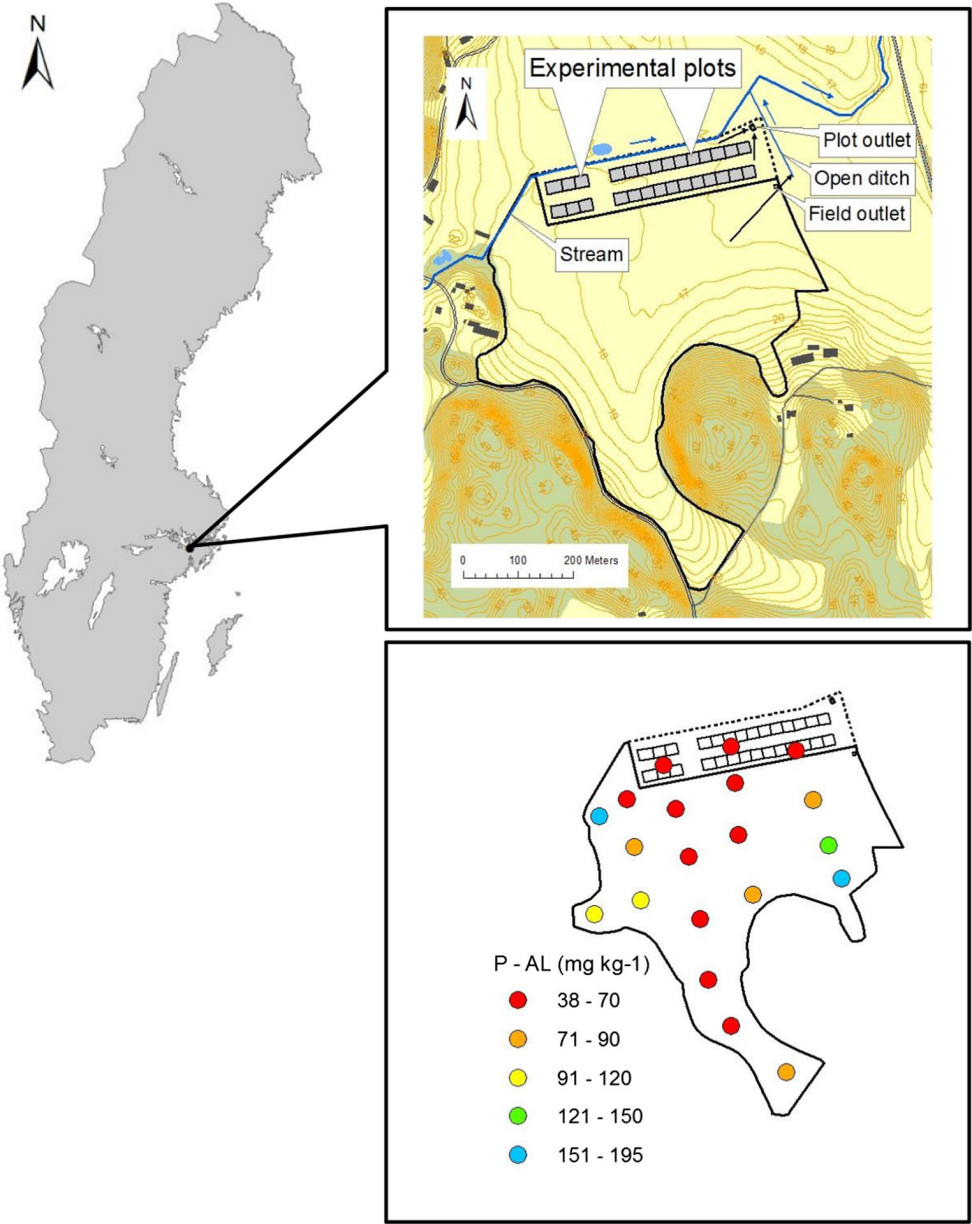
Oxelby experimental plots (southern and northern row of 14 drained plots) and Oxelby field, with ammonium lactate-extracted topsoil phosphorus concentrations (P-AL). The water from the field and from the plots runs to an open ditch in the centre of the small valley. A small stream runs parallel to the plot rows

The Oxelby experimental site, is located in eastern Sweden, encompassing an area with drained plots (the *Oxelby plot experiment*) and an adjacent observation field (the *Oxelby field*) (Fig. 1). Leaching experiments are combined with measured yields at both sites. The experimental set-up of the plot experiment, with each plot having a separate subsurface water collection system, was constructed in 2006 (Svanbäck et al. 2014). There are 28 plots, situated in two rows of 14, in a small, nearly flat valley (mean slope < 0.5%) with downslope cutoff subsurface drains towards the Oxelby field (mean slope < 1%). There is a similar area with cutoff drains between the plot rows. The southern row borders the Oxelby field (Fig. 1), while the northern row is 40 m closer to a small stream running parallel to the rows than the southern row. The water from the plots and from the field enters a large open ditch situated in the centre of the valley.

#### The Oxelby plot experiment

Each Oxelby plot (size 20 m × 24 m) has subsurface drains placed centrally at 1 m depth and with 8 m spacing. There is a coarse gravel (8–16 mm) layer on the drainage ditch base and around and above the drain pipes to approximately 50 cm depth from the soil surface. The individual plot borders are separated with plastic sheeting below ploughing depth (30 cm). Mean soil P-AL was 32 mg kg^−1^ in 2006, with low spatial variation (Svanbäck et al. 2014). Topsoil texture is silty clay, with the clay content generally increasing towards the centre of the valley.

**Fig. 2:**
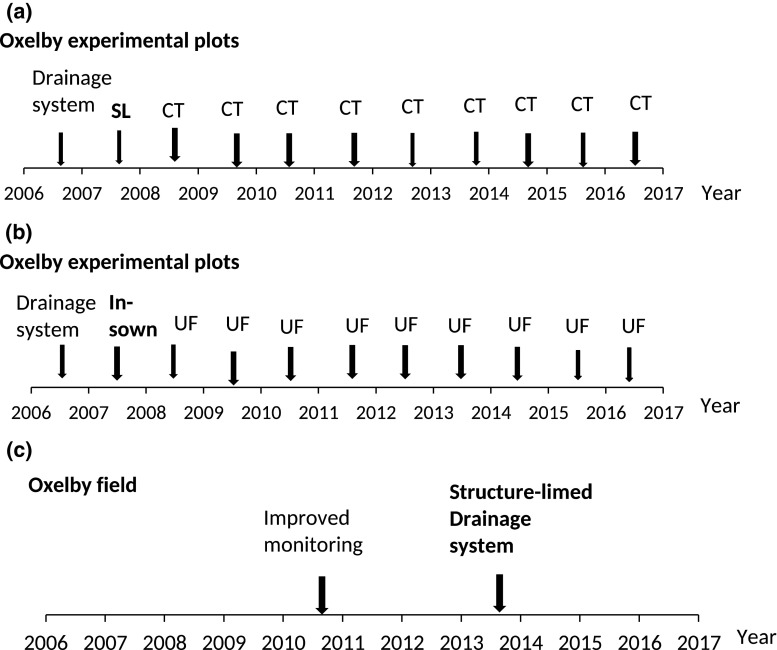
Timelines for measures: (*a*) experimental structure-limed plots followed by conventional tillage each autumn (SL-CT); (*b*) fallow sown in first year and with the grass cut yearly (UF); (*c*) Oxelby field structure-limed, the drainage system renovated and lime incorporated into the backfill above the drains

The plots were treated with different P mitigation options in four randomly placed replicates. The four soil management options (16 plots) assessed here were conventional tillage by ploughing (CT); liming in the form of quicklime (CaO) in the first year (2007) (Fig. 2a) followed by conventional tillage (SL-CT); yearly reduced tillage (ShT) and unfertilised fallow (UF) sown in the first year (Fig. 2b). All treatments were applied consistently for eight years. Treatment CT represents the most common practice for the region, with mouldboard ploughing inverting the soil to about 23 cm depth in late autumn. In treatment SL-CT, the quicklime was applied in dry conditions to stubble at a rate of 5 t CaO ha^−1^ and immediately mixed into the soil using a cultivator to the depth of 15 cm. For treatment ShT, plots were tilled to 12–15 cm depth with a cultivator twice in all years and reconsolidated with a rib-roller in 2010–2012. For the UF treatment, the grass was cut yearly and left on the plots. Spring-sown crops (barley, oats and field peas) which allowed annual autumn tillage were used in all treatments except UF, (Svanbäck et al. 2014). All treatments (except UF) received the recommended P fertiliser dose to replace the amount removed with the harvested crop.

#### The Oxelby field

The soil at the Oxelby field (Ulén and Persson 1999) is silty clay with spatially variable soil P status (mean in 2006 70 mg kg^−1^ P-AL), representing low concentrations in the main lower part of the field and higher concentrations in the upper part (Fig. 1). The soil is artificially drained, with old tile drains running to a collection pipe ending 110 m from the outlet of the experimental plots, where drainage and P concentrations are monitored. The field was conventionally ploughed for cash crops or has been under ley (for crops, tillage and fertilisation in the period 2010–2016, see Table 1). Only small amounts of mineral P fertiliser has been applied, since P application is restricted in the area because the recipient, Lake Bornsjön, is a reserve drinking water reservoir for Stockholm city. In August 2013 (Fig. 2c), the entire field area was structure-limed with a commercial mixture containing slaked lime [Ca(OH)_2_], at a rate equivalent to 1 ton ‘active’ CaO ha^−1^. The lime was applied in dry weather immediately followed by incorporation by two passes of a cultivator in different directions to the depth of 12 cm. Three weeks later, the drainage system was renovated with drainage pipes placed in a herring-bone pattern at 14 m spacing (> 0.07 m m^−2^) in the entire middle and lower part of the field (two-thirds of the area), while the uppermost one-third of the field was left without drain renovation. Gravel (4–8 mm) was placed in the bottom of the drains, which amounted in total to 15 375 m. Structure lime containing 18% active CaO, equivalent to 1.2 kg CaO m^−1^, was incorporated by two passes of a cultivator into the soil used as backfill above the drains in the more central part of the field (corresponding to another one-third of the field area). The backfill in the lowest part of the field (corresponding to still another one-third of the field area) and having the highest clay content was treated with higher intensity and received double the amount of structure lime.

**Table 1:** Crop, date of tillage and phosphorus fertilisation of Oxelby field in six agrohydrological years (2010–2016). No manure P was applied

Year	2010/2011	2011/2012	2012/2013	2013/2014	2014/2015	2015/2016
Crop summer	Ley	Winter wheat	Barley	Fallow	Winter wheat	Winter wheat
Crop winter	Winter wheat	–	Fallow	Winter wheat	Winter wheat	–
Date of tillage I^a^	2010-08-28^a^	2011-08-20^b^	–	2013-08-20^b^	2014-08-19^b^	2015-08-30^b^
Date of tillage II	2010-12-02^a^	2011-09-23^b^	–	2013-08-20^b^	2014-09-08^b^	–
Mineral P (kg ha^−1^)	10	15	10	–	9^c^	9^c^
Date applied	2010-04-19	2011-04-26	2012-05-17	–	2014-04-15	2014-04-11

^a^Conventional ploughing

^b^Only two passes with a cultivator, in autumn 2013 in connection with structure liming

^c^Mineral fertiliser with slow release of P

### Precipitation, water discharge, water sampling and water analysis

Precipitation was monitored at Södertälje, about 6 km south-east of the experimental sites. Water discharge from the Oxelby plot experiments was measured with tilting vessels in an underground measurement basement. For the Oxelby field, water discharge was measured in a large concrete well in or beside a monitoring cabin, where water drainage flow was gauged continuously through an open V-notch weir (90°) with the water level calibrated to a displacement body acting as a float and hanging in a load cell connected to a datalogger.

Flow-proportional sampling of drainage water was carried out at both sites. Each flow-proportional subsample represented a certain amount of discharge, typically 0.04 L m^−2^ for the plots and 0.15 L m^−2^ for Oxelby field. Composite water samples were collected in glass vessels (2.5 or 10 L) at approximately 10–14 °C in darkness for a maximum of one week. The analysis from the plots mainly took place during periods of turbid water but not in periods of clear water or when small amounts of water were collected. The water samples were immediately sent for chemical analysis to the Water Laboratory at the Department of Soil and Environment, SLU (after spring 2014 the Department of Water and Environment, SLU). They were transported in 100 mL glass bottles until the transfer to the new laboratory in spring 2014, when plastic bottles were used. Dissolved reactive P (DRP) was analysed (EN ISO 2003) within two days after storage at +4 °C and after pre-filtration using filters with a pore diameter of 0.2 μm (Schleicher & Schüll GmbH, Dassel, Germany). Total P (TP) was analysed within 4 days after storage +4 °C as DRP and after acid oxidation with K_2_S_2_O_8_ (ISO 2003). Particulate P (PP) was estimated as the difference between TP in filtrated and unfiltrated samples from the Oxelby field. From this site, suspended solids (SS) were also analysed by weighting the filter-cake on the same filters as used for pre-filtration of the DRP filters. Turbidity (Turb) was determined immediately after shaking the sample and by turbidimeter (Hach-Lange Company, Düsseldorf, Germany) from the Oxelby field 2010–2016 and likewise in later years (2014–2016) from the other plots. Unreactive P (UP), estimated as the difference between TP and DRP, was used as a proxy for particle-bound P (PP) and the ratio of UP to turbidity for P content in water particles from the experimental plots.

### Data analysis: Statistics and estimations

Yearly leaching was estimated from the flow-proportional composite samples. Discharge, leaching and flashiness index were estimated based on ‘agrohydrological years’ (1 July–30 June). The flashiness index was calculated for each year using the Richards–Baker formula modified to account for hourly fluctuations in drain discharge:

$$ {\text{FI}}_{\text{hour}} \frac{{\mathop \sum \nolimits_{i = 1}^{n} \left| {(q_{\text{i}} - (q_{i - 1} )} \right|}}{{\mathop \sum \nolimits_{i = 1}^{n} q_{\text{i}} }}, $$

where *q*_i_ is flow in hour *i*, *q*_i−1_ is flow in the previous hour and $\sum\nolimits_{i = 1}^{n} {q_{i} }$ is total flow in one year. The FI_hour_ value obtained was in the present study multiplied by 24 in order to facilitate comparisons with other leaching studies using the more common FI_day_ index.

A general linear mixed model (ANCOVA, SAS software Version 9.2) was used to analyse differences in P leaching between the different treatments in the Oxelby experimental plots. To account for the time series structure of the data, correlations between measurements over time were modelled with AR (1) correlation structure (Littell et al. 2006). Treatment was used as fixed factor, while distance to the centre of the valley was used as covariate. In parallel, FI_hour_ was tested as covariate, assuming that it was unaffected by treatment. A significance level of *α* = 0.05 was used, including the probability coefficient (*p*) value for differences, adjusted according to the Tukey–Kramer multiple comparison procedure. Pearson correlation and *p* coefficient were used as measures of the degree of dependency and coefficient of determination in regression analysis as a measure of the direction of any dependence. Graphical assessment with leaching related to both FI_hour_ and *Q* was performed for Oxelby field with a short (three-year) post-period after treatment.

## Results and discussion

### Precipitation drain discharge and flashiness index

#### The Oxelby plot experiment

Precipitation at Södertälje was c. 660 mm year^−1^ for the study period. In summer in particular, the drains leading from the plots dried out or only contained visually clear water with low turbidity. Yearly discharge varied between 200 and 900 mm year^−1^ and the mean *Q*/Prec ratio was 0.70, but *Q* was actually higher than Prec for two of the plots situated in the north row.

**Fig. 3:**
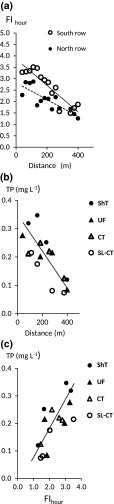
**a** Mean (eight-year) water flow index (FI_hour_) related to distance to the central ditch for the south and north row for each plot; **b** mean flow-weight total phosphorus (TP) concentration for each of the 16 plots related to the same distance with yearly reduced (shallow) tillage (ShT), unfertilised fallow (UF), conventional tillage (CT) and structure liming in the first year followed by conventional tillage (SL-CT) and **c** the same TP concentration related to FI_hour_ with a regression line. All relationships showed significant correlations (*p* < 0.05). The slope of the relationship between (FI_hour_) and distance was 1.6 times higher for the south row than for the north row. Distance as a function of TP concentration had coefficient of determination (*r* ^2^) = 0.52 and FI_hour_ as a function of the same concentration had *r* ^2^ = 0.50

Mean FI_hour_ for the plots was 2.34 and was on average three-fold higher than the corresponding FI_day_ value. The spatial variation in FI_hour_ in the study years (coefficient of variation (CV) = 42%) was higher than the temporal variation (CV = 35%). The flow index for treatments increased in the order UF > CT > ShT > SL-CT (Table 2), but with no significant differences. The same index was significantly (*p* < 0.001) negatively correlated with *Q*. There was a significant difference in *Q* (*p* < 0.001) between rows, with the southern plot row having lower mean discharge (*Q* = 393 mm year^−1^) and higher mean FI_hour_ (2.67) than the northern row located closer to the stream (*Q* = 514 mm year^−1^; mean FI_hour_ = 2.06). Correspondingly, the slope of the plot between FI_hour_ and distance from the stream was higher for the southern plot rows (Fig. 3a). Proximity to the small stream is probably the main explanation for the differences between the two rows of plots. Flooding by the stream was not observed, but the stream probably influenced drain discharge. This might have led to prolonged, but damped, water flow pulses from plots in the north row.

**Table 2:** Mean precipitation (Prec), discharge (Q), Q/Prec ratio, yearly hourly based water flow index (FI_hour_), yearly transport of suspended solids (SS) total phosphorus (TP) and dissolved reactive P (DRP) and yearly flow-weighted concentrations of TP in agrohydrological years from experimental drained plots representing shallow autumn tillage (ShT), unfertilised fallow (UF), conventional autumn tillage (CT) and structure liming in 2007, followed by conventional autumn tillage (SL-CT) over an eight-year period. The same parameters for Oxelby field (representing a crop rotation given in Table 1) in three years before and after combined measures. Last row refers to the factor distance ‘to the valley centre’ as a mean for plots with different treatments

Period	Experimental plots				Oxelby field	
	2007/2015				2010/2013	2013/2016
Treatments	ShT	UF	CT	SL-CT	Crop rotation	
Prec. (mm year^−1^)	680	680	680	680	691	640
Q (mm year^−1^)	511	449	421	501	368	315
Ratio Q/Prec	0.75	0.66	0.62	0.74	0.53	0.49
FI_hour_	2.23	2.45	2.31	2.14	1.59	1.73
SS (kg ha^−1^ year^−1^)	–	–	–	–	650	302
TP (kg ha^−1^ year^−1^)	1.21^ab^	0.84	0.81	0.57	1.00	0.45
DRP (kg ha^−1^ year^−1^)	0.18	0.15	0.13	0.13	0.20	0.13
TP (mg L^−1^)	0.24	0.19	0.19	0.11	0.28	0.15
Mean distance (m)	220	226	220	232	–	–

^ab^ShT tended to be higher than SL-CT

^a^With FI hour as covariate, Pr > F 0.084; adjusted *p* = 0.060 (Tukey–Kramer)

^b^With distance as covariate, Pr > F 0.057; adjusted *p* = 0.009 (Tukey–Kramer)

The FI_hour_ values decreased with the distance to the ditch (Fig. 3a) and were 1.7–2.8 times as high close to the centre of the valley than at points 400 m away. The FI_hour_ values also increased with increasing topsoil clay concentration (coefficient of determination *r* ^2^ = 0.74 *p* < 0.05). Since high clay content is often associated with a high susceptibility to preferential flow (Koestel et al. 2012; Koestel and Jorda 2014), this strong correlation suggests that non-random spatial differences in topsoil and subsoil structure were the main reason for the large spatial variation in FI_hour_. Random differences in the backfill of the drainage system may further explain the different hydrological signatures for different plots, since macroporosity around the actual drain is known to have a strong effect on drain discharge dynamics (Alakukku et al. 2010). The concentration of the weakly adsorbed herbicide bentazon in drainage water after application on June 2011 was significantly related to FI_hour_ in the actual year (2010/2011) (data not shown). This relationship was more apparent for the southern than the northern row, but for both rows the *r* ^2^was high (0.76).

#### The Oxelby field

Mean discharge from the Oxelby field (341 mm year^−1^) was lower than from the plots (475 mm year^−1^) in the measurement periods 2010–2016 and 2010–2015. The corresponding FI_hour_ values were generally lower (mean 1.66), for Oxelby field than for the plots (mean 2.28). For Oxelby field, mean *Q* and *Q*/Prec (Table 2) were not significantly different in the three-year period after and the three-year period before renovation of the drainage system and application of structure lime to the backfill. Changes in infiltration rate after improving the drainage system were not accompanied by a dramatic change in discharge amount, which was slightly lower in the post-period compared with the pre-period (Table 2). Yearly FI_hour_ was 1.17–1.77 in five out of six years, but was unusually high (2.84) in 2014/2015 (post-period). Based on the limited number of years with water drainage flow gauged by a displacement body and with automatic flow-proportional sampling (2010–2016), the relationship between FI_hour_ and discharge over time was unclear, but the parameters tended to be positively correlated (*p* = 0.06).

### Long-term concentrations and leaching of phosphorus related to treatments

#### The Oxelby plot experiment

Total P concentrations were significantly negatively correlated to the distance to the valley centre for the means of all eight years (Fig. 3b). In that diagram, the regression line illustrates flow-weighted mean concentrations for all four treatments, with all plots representing ShT situated above and all mean concentration from CT below the regression line. In addition, TP concentration was significantly positively correlated to FI_hour_ (Fig. 3c).

Mean yearly leaching of P is presented in Table 2. Only a minor part was in dissolved form (16%) except in treatment SL-CT, where DRP constituted 23% of TP leaching. Leaching of TP for all eight years of observation was only correlated to Q for treatments ShT and UF (*p* < 0.05), and not for CT and SL-CT (*p* = 0.36). Hence Q could not be used as a predictor when comparing liming with the effect of other treatments. Treatment effects were not significant (*p* = 0.084) when using Richards–Baker flow index as covariate. A similar result was obtained using distance as covariate (*p* = 0.057). A tendency for lower leaching was obtained comparing SL-CT with ShT (*p*_adj_ = 0.060 and 0.009, respectively). No significant differences or tendencies were found for DRP leaching between different treatments. Lower TP leaching with SL-CT compared with other treatments was not observed in this extended study which is in contrast to the results presented by Svanbäck et al. (2014) which covered six years and three more treatments. Further monitoring is needed to confirm any trend for reduced efficiency, e.g. from successively accumulation of soil P overtime without inverting the soil as discussed by Dodd and Sharpley (2016) or reduced long-termed effect of structure liming. Biological factors, e.g. a larger earthworm population promoted by liming compared to non-liming should also be investigated since earthworm’s activities may have a profound effect on biopores and P leaching (e.g. King et al. 2015).

**Fig. 4:**
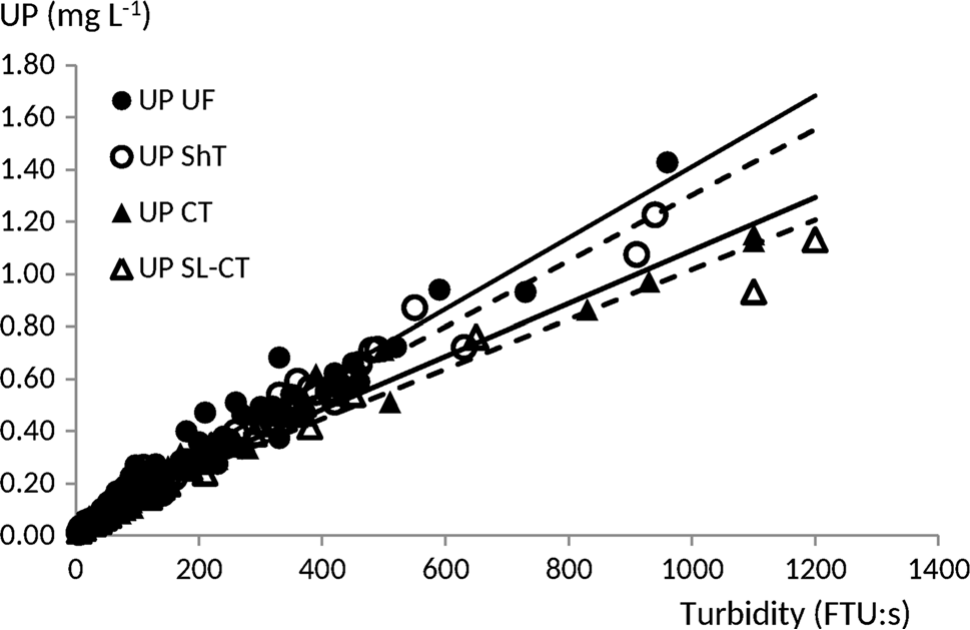
Concentration of unreactive P related to turbidity from unfertilised fallow (UF), shallow tillage (ShT), conventional tillage (CT) and structure-limed followed by conventional tillage (SL-CT) in agrohydrological years 2014–2015. Coefficient of determination was estimated to be 0.94–0.97. Unreactive phosphorus (UP)-to-turbidity ratio was significantly higher (*p* < 0.05) for UF and ShT than for CT and SL-CT

Turbidity can be assumed to be a good proxy for SS from the plots, since this relationship had a high coefficient of determination (*r* ^2^ = 0.95) in drainage water from Oxelby field, where both parameters were analysed simultaneously. Erosion of SS from CT and UF plots was approximately 500 kg ha^−1^ year^−1^ on average over seven years based on the turbidity values in later years. Unreactive P from the plots was significantly correlated to turbidity, but with different slopes of the regression line for different treatments (Fig. 4). The ratio of UP to turbidity was significantly higher for the UF and ShT plots than the CT and SL-CT plots, suggesting that not inverting the soil by ploughing had resulted in a higher P content in the solid phase of the topsoil. This was also indicated by the P contents in a few topsoil samples collected in autumn 2012 (data not shown). Long-term studies of untilled plots are advisable since macropore flow may become enhanced due to higher macroporosity beside a P-enrichment of the topsoil particles compared to conventionally ploughed plots. The overall results also demonstrate that large numbers of replicates are needed in P mitigation leaching studies in order to ensure that natural variations in both space and time are covered and that any gradual changes, e.g. redistribution of topsoil P taking place as a result of plant root growth, can be detected.

### Phosphorus leaching before and after measures at Oxelby field

**Fig. 5:**
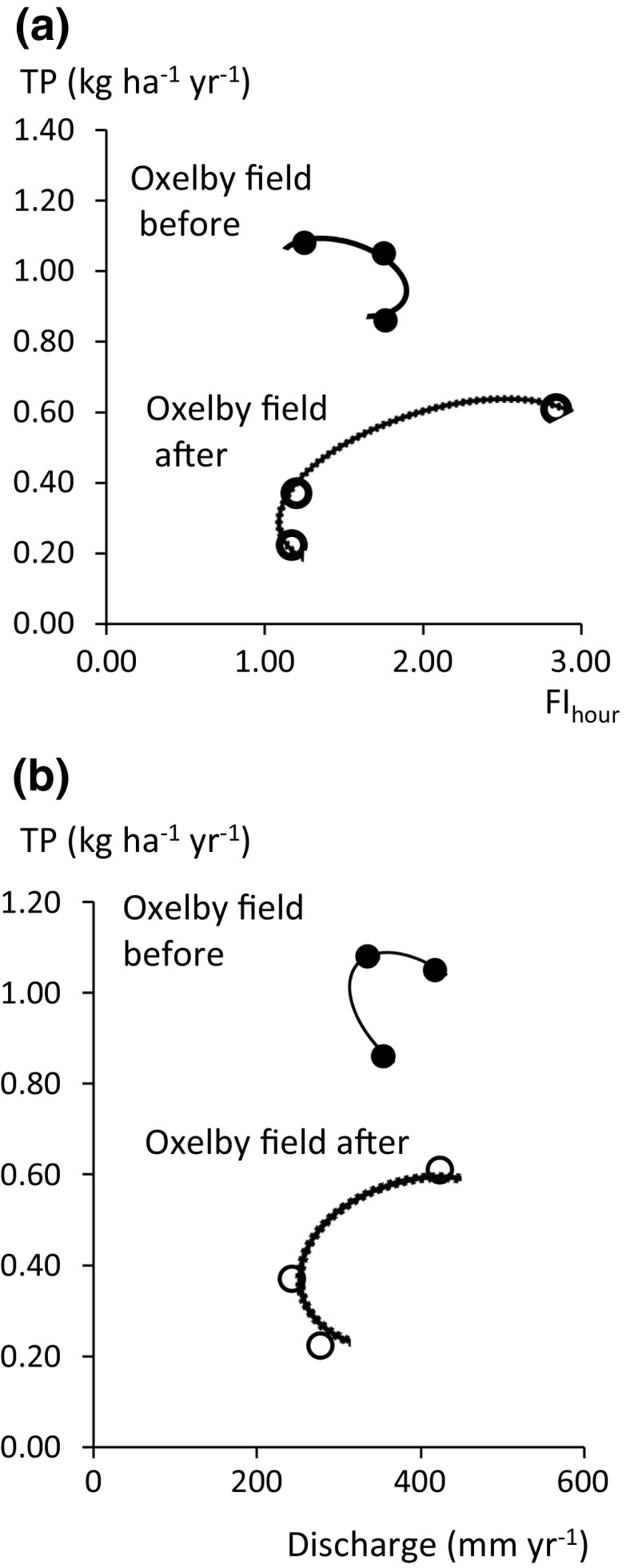
Yearly leaching of total P (TP) related to: **a** flow index (FI_hour_) and **b** discharge (*Q*) for Oxelby field before (filled circles) and after (unfilled circles) combined measures

Phosphorus application with mineral fertiliser (9–15 kg P ha^−1^ year^−1^) (Table 1) was always less than average removal with the crop (16 kg ha^−1^ year^−1^) based on measured yields from the field and crop P concentrations in nearby Oxelby plot experiment (Svanbäck et al. 2014). High yields of winter wheat (7.2 and 8.4 t ha^−1^) were recorded in 2014 and 2015 and more P (mean 15 kg ha^−1^ year^−1^) was removed with the crop than applied with P fertiliser. Well-balanced fertilisation utilising soil P reserves has been demonstrated to reduce P leaching compared to former over-fertilisation, but at a very slow rate and with an initial lag phase (Ulén et al. 2015). Graphical assessment strongly indicated a reduction in TP leaching both in relation to FI_hour_ and *Q* (Fig. 5a, b). The prompt reduction in leaching at the Oxelby field was found also for SS, and PP (Table 2), hence suggests an effect of the renovation of the drainage system, introduction of lime-filter ditches and field application of structure liming on these parameters. Phosphorus transport as TP and PP was 40–50% lower, whereas water discharge was only 14% lower in the second period. Mitigation of DRP leaching seemed also to have taken place, as flow-weighted mean DRP concentration decreased from 0.20 in 2010/2013 to 0.13 mg L^−1^ in the following year. This observation is in agreement with monitoring data for the outflow of new drainage systems installed in 1989 in Lithuania, where average DRP concentration was 40% lower with lime-filter backfill compared with zero treatment (Bastiene et al. 2012). For Oxelby field, flow-weighted mean TP concentration decreased from 0.28 to 0.15 mg L^−1^ based on the entire three-year pre- and post-periods.

## Conclusions

Spatial variation in phosphorus leaching via subsurface drains was higher than the temporal (eight-year) variation at the Oxelby experimental site with gradients in clay soil content and plots with varying positions in the valley. Thus large numbers of replicates are needed in phosphorus leaching studies to ensure that such natural spatial variation is covered. Here the FI_hour_ index used indicated an even more varied spatial P leaching than in the formerly P leaching assessment using the site-specific factor of distance to the valley centre. However, FI_hour_ may have been affected by treatments designed to improve soil structure thus changing this hydrological signature from, e.g. SL-CT. Both factors gave similar results when used separately as predictors of phosphorus concentrations and water transport.

Long-term studies of untilled and shallow tillage plots are required, since the particles in drain water from these treatments were observed to be more P-enriched than particles from conventionally ploughed plots and since macropore flow may be enhanced in untilled soil due to higher macroporosity.

Combined treatment to improve drainage and water infiltration in different parts of fields, as tested here, seems to be a promising strategy for mitigating phosphorus leaching but any effects from the different treatments cannot be separated based on monitoring results from a single field. Long-term monitoring is advisable in order to quantify P leaching effects particularly from such a field, treated in several ways.

## References

[CR1] Alakukku L, Nuutinen V, Ketoja E, Koivusalo H, Paasonen-Kivekas M (2010). Soil macroporosity in relation to subsurface drain location on a sloping clay field in humid climatic conditions. Soil and Tillage Research.

[CR2] Baker DB, Richards RP, Timothy T, Loftus TT, Kramer JW (2004). A new flashiness index: characteristics and applications to Midwestern rivers and streams. Journal American Water Resource Association.

[CR3] Bastienė, N., V. Šaulys, and V. Gurklys. 2012. An assessment of lime filter drainage systems. In: *Drainage systems*. M.S. Javald, 181–210. InTech: Retrived 5 September 2016 from http://www.intechopen.com/books/drainage-systems/an-assessment-of-lime-filter-drainage-systems.

[CR4] Beven K, Gerdman P (2013). Macropores and water flow in soils revisited. Water Resources Research.

[CR5] Deelstra J, Iital A (2008). The use of the flashiness index as a possible indicator for nutrient loss prediction in agricultural catchments. Boreal Environment Research.

[CR6] Deelstra J, Iital A, Povilaitis A, Kyllmar K, Greipsland I, Blicher-Mathiesen G, Jansons V, Koskiaho J (2014). Hydrological pathways and nitrogen runoff in agricultural dominated catchments in Nordic and Baltic countries. Agriculture Ecosystem and Environment.

[CR7] Dodd RJ, Sharpley AN (2016). Conservation practice effectiveness and adoption: unintended consequences and implications for sustainable phosphorus management. Nutrient Cycling in Agroecosystems.

[CR8] Egnér H, Riehm H, Domingo WR (1960). Investigations on chemical soil analysis as the basis for estimating soil fertility. II. Chemical extraction methods for phosphorus and potassium determination. Kungliga Lantbrukshögskolans Annaler.

[CR9] EN ISO 2003 6878 Water Quality Determination of phosphate. Ammonium molybdate spectrometric method. Retrieved 5 September 2016 from http://www.sis.SR/Templates/SIS.

[CR10] Elmquist T (2014). Drainage of agricultural land. Swedish Official statistics. JO 10 SM 1401.

[CR11] Haygarth PM, Condron LM, Heathwaite AL, Turner BL, Harris GP (2005). The phosphorus transfer continuum: Linking source to impact with an interdisciplinary and multi-scaled approach. Science of the Total Environment.

[CR12] ISO 15681-1. 2003: Water quality—determination of phosphate and total phosphorus by flow analysis (CFA and FIA). Part 1: Method by flow injection analysis (FIA). International organisation for Standardization. Retrieved 5 September 2016 from http://www.iso.org/.

[CR13] Jarvis N (2007). A review of non-equilibrium water flow and solute transport in soil macropores: Principles, controlling factors and consequences for water quality. European Journal of Soil Science.

[CR14] King KW, Williams MR, Macrae ML, Fausey NR, Frankenberger J, Smith DR, Kleinman PJA, Brown LC (2015). Phosphorus transport in agricultural subsurface drainage: A Review. Journal of Environmental Quality.

[CR15] Koestel JK, Moeyes J, Jarvis NJ (2012). Meta-analysis of the effects of soil properties, site factors and experimental conditions on solute transport. Hydrology and Earth System Sciences.

[CR16] Koestel JK, Jorda H (2014). What determines the strength of preferential transport in undisturbed soil under steady-state flow?. Geoderma.

[CR17] Lindström J, Ulén B (2003). The impact on CaO in the backfills on P losses from arable land. Report in Swedish to the Swedish Board of Agriculture.

[CR18] Littell R, Milliken G, Stroup W, Wolfinger R, Schabenberger O (2006). SAS for mixed models.

[CR19] Norgaard T, Moldrup P, Olsen P, Vendelboe V, Greve MH, Kjær J, de Jonge LW (2013). Comparative mapping of soil physical-chemical and structural parameters at field scale to identify zones of enhanced leaching risk. Journal of Environmental Quality.

[CR20] Šaulys V, Bastienė N (2007). The impact of lime on water quality when draining clay soils. Ekologija.

[CR21] Skaggs RW, Brevé MA, Gilliam JW (1994). Hydrologic and water quality impacts of agricultural drainage. Critical Reviews in Environmental Science and Technology.

[CR22] Svanbäck A, Ulén B, Etana A (2014). Mitigation of phosphorus leaching losses via subsurface drains from a cracking marine clay soil. Agriculture, Ecosystems & Environment.

[CR23] Taylor SD, He Y, Hiscock K (2016). Modelling the impact of agricultural management practices on river water quality in eastern England. Journal of Environmental Management.

[CR24] Ulén B, Persson K (1999). Field-scale phosphorus losses from a drained clay soil in Sweden. Hydrological Processes.

[CR25] Ulén B, Larsbo M, Kreuger J, Svanbäck A (2013). Spatial variation in herbicide leaching from a marine heavy clay soil via subsurface drains. Pesticide Management Sciences.

[CR26] Ulén B, Johansson G, Kyllmar K, Stjernman Forsberg L, Torstensson G (2015). Lagged response of nutrient leaching to reduced surpluses at the field and catchment scales. Hydrological Processes.

[CR27] Ulén B, Stenberg M, Wesström I (2016). Use of a flashiness index to predict phosphorus losses from subsurface drains on a Swedish farm with clay soils. Journal of Hydrology.

